# Introduction to Maxxam All-Season Passive Sampling System and Principles of Proper Use of Passive Samplers in the Field Study

**DOI:** 10.1100/tsw.2001.80

**Published:** 2001-09-24

**Authors:** Hongmao Tang

**Affiliations:** Centre for Passive Sampling Technology, Maxxam Analytics Inc., Edmonton, Alberta, Canada T6B 2R4,

**Keywords:** passive sampling technology, air pollution, sulfur dioxide, nitrogen dioxide, ozone, hydrogen disulfide

## Abstract

Maxxam all-season passive sampling system (PASS) is introduced in this paper. The PASS can be used to quantitatively and accurately monitor SO_2_ , NO_2_, O _3_, and H_2_ S in air in all weather conditions with flexible exposure times from several hours to several months. The air pollution detection limits of PASS are very low. They can be from sub ppb to ppt levels. The principles of proper use of passive samplers in the field study are discussed by using the PASS as an example.

## INTRODUCTION

Scientific and social interest in monitoring air pollutants indoors and outdoors is manifest in the world. Many monitoring technologies for $SO_2$, $NO_2$, $O_3$, and $H_2S$ in air have been developed and subsequently improved in the past few decades. Generally speaking, these technologies can be classified as integrative collection and real-time analytical technologies. Both technologies can be further divided into two categories: active and passive methods.

Active methods directly pump air through collection or analytical devices to collect or analyze air pollutants. Therefore, electrical power, roads or shelters are required. A passive (or diffusive) sampler is a device which is capable of taking samples of gas or vapor pollutants from air at a rate controlled by a physical process such as diffusion through a static air layer or permeation through a membrane. However, it does not involve the active movement of the air through the sampler. Passive samplers are generally simple in structure and do not require electricity and thus are cost effective and convenient to use.

The key parameter related to the correct measurement of air pollutants by using passive samplers is the sampling rate. Active samplers have a known sampling rate, which is the pump's flow rate. The passive sampler's sampling rates depend on many factors such as temperature, relative humidity (RH), wind direction, wind speed, sampler's structure, and collection media. If a passive sampler can be used in all climate conditions, a fixed passive sampling rate obtained from laboratories cannot be used for ambient studies anywhere and anytime in the world. It would be highly unreasonable to expect that a passive sampler's sampling rate would be the same when temperatures change from $-30^o$ to $+30^oC$ and relative humidities change from 90 to 15%. Therefore, the key factor for using passive samplers is how to determine their sampling rates.

Commonly used passive sampling devices for $SO_2$ include a lead dioxide candle (Pb-CAD), Huey plate (H-plate), and $K_2CO_3$ plate (K-plate). It has been determined that there are deficiencies with these existing $SO_2$ passive samplers[1]. Several groups also reported different $SO_2$ passive samplers[2,3]. Unfortunately, fixed sampling rates obtained by laboratory are used by the authors.

Different $NO_2$ passive samplers have been developed. Typically, triethanolamine (TEA) is used as a collection medium in the passive samplers since it captures these pollutants very efficiently, and ion chromatography (IC) can be used to conduct analysis with a low detection limit. Scientists in the U.S. have investigated the performance of nitration plates using controlled test atmospheres[4]. Their studies found that temperature, relative humidity and $NO_2$ concentration strongly affected the nitration plate sampling rates. Scientists from Europe found that TEA could not be used for long-time sampling, e.g., several weeks[5]. Their studies revealed that passive samplers covering the whole period always gave lower results than the summary of the successive samplers.

Ozone ($O_3$) passive samples have also been troublesome until recently. One reason is that the collection media used to sample ozone also reacts with other oxidants in the atmosphere such as peroxy acyl nitrates and oxides of nitrogen and sulfur. Another reason is the design of the passive samplers using an air gap as a diffusion barrier. For outdoor use, these kinds of passive samplers will be affected by wind direction, wind speed, and dust[6].

An $H_2S$ passive sampler, in common use for many years utilizes a paper impregnated with zinc acetate[7]. The detection limit of $H_2S$ using this method is quite high (2 mg/l in the test solution), and interference from $SO_2$ in the atmosphere is inevitable. Therefore, the accuracy for this method is questionable. Like other inadequate passive methods, the zinc acetate paper is directly exposed to the atmosphere without any protection such as a diffusion barrier. The diffusion barrier serves to eliminate the effects of particulate contamination and also makes it possible to account for the variables of wind speeds which affect the sampling rate of the device.

It is clear that although passive sampling technology is simple and cost effective compared to active methods, it might be difficult to obtain accurate results if the passive sampler is not designed and used properly. A new Maxxam all-season passive sampling system (PASS) is introduced in this paper. Principles of proper use of passive samplers in the field study are discussed by using the PASS as an example.

## MAXXAM ALL-SEASON PASSIVE SAMPLING SYSTEM

The Centre for Passive Sampling Technology of Maxxam Analytics Inc. (CPST) has successfully developed an all-season Passive Air Sampling System (PASS) for sampling $SO_2$, $NO_2$, $O_3$, and $H_2S$ in the atmosphere (see Fig. 1)[6,8,9,10,11] with the support of Alberta Environmental Protection (AEP), Alberta Research Council (ARC), Clean Air Strategic Alliance of Alberta (CASA) and the National Research Council of Canada (NRC). The PASS has passed several independent validations from different government organizations in Canada. The special features of the PASS are as follows.

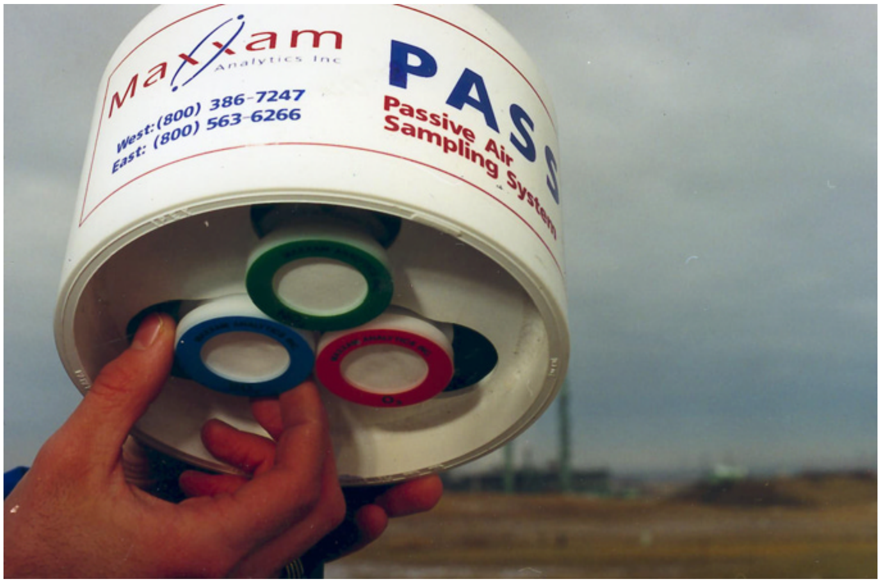

**FIGURE 1.** Passive samplers of Maxxam all-season passive sampling system.

* The PASS includes four elements:

1. A newly designed passive sampler body (Fig. 1).
2. A newly designed rain shelter (Fig. 2). This rain shelter allows the passive sampler be installed face downwards outdoors.
3. Specially selected and developed collection media.
4. Equations to calculate sampling rates for different air pollutants. The equations account for variations in temperature, relative humidity, and wind speed.

The downward facing installation of the passive samplers can prevent the precipitation of suspended particles in the atmosphere on the surface of the diffusion barriers. This prevents the air pollutants from being absorbed by the particles and avoids the pore-size change of the diffusion barrier, which will eventually change the sampling rate. Face downward installation also helps to obtain air movement across the diffusion barrier surface of a passive sampler, which is the most sufficient way to reduce the air pollution concentration boundary layer generated during sampling.

* The PASS is very sensitive. It can be used to collect ppt levels of $H_2S$ and sub ppb levels of $SO_2$, $NO_2$, and $O_3$ in air.

* The collection time is very flexible. It can be exposed from several hours to several months depending on the monitored pollutant and its average concentration in air.

* A new collection medium for sampling $NO_2$ — CHEMIX™ — has been developed. CHEMIX™ has been shown to be a highly efficient collection medium for $NO_2$ in air. Compared to TEA, it is less affected by temperature and relative humidity, and has a higher sampling rate[10].

* By attaching a clip to the sampler body, the PASS sampler can be used as a personal monitor (Fig. 3).

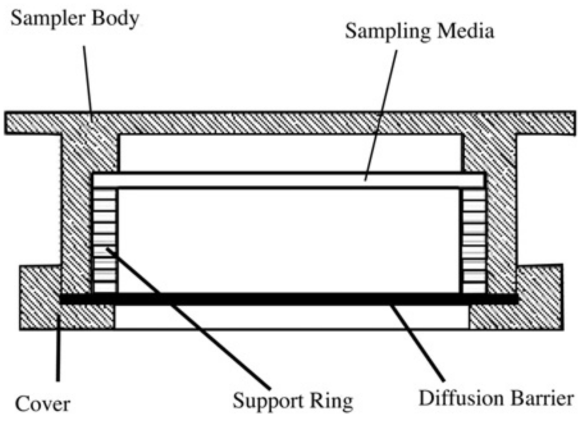

**FIGURE 2.** Rain shelter of Maxxam all-season passive sampling system with three passive samplers.

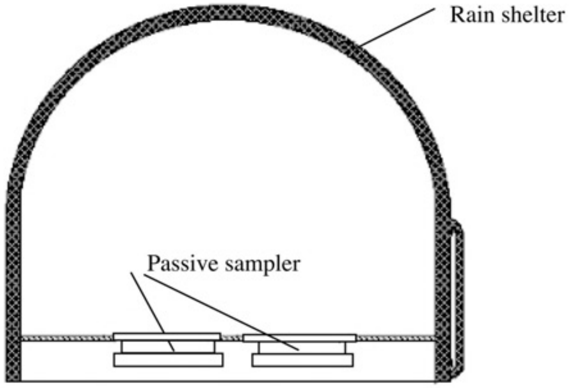

**FIGURE 3.** Personal monitor of the PASS $H_2S$.

## Laboratory Study

The PASS was developed in a laboratory passive study system in the CPST. The study temperatures ranged from –37° to 32°C, relative humidities from 4 to 95%, and wind speed from 0.5 to 150 cm/s. Sampling rate equations were first derived from the laboratory studies, then validated in field studies. Different air pollutants have different equations[6,8,9,10,11]. The following is an example for calculating PASS $SO_2$ sampling rate[9].

$$R_S = 12.769T^{1/2} - 0.540RH + 0.276WSP - 135 \qquad (1)$$

where T = average temperature, K, RH = average relative humidity[5] (if RH > 80, then RH = 80), and WSP = average wind speed, cm/sec (if WSP > 130, then WSP = 130). The average temperature, relative humidity, and wind speed can be obtained from local weather stations if the monitoring station cannot provide the information. From Equation 1, it can be seen that the PASS passive sampler performance is proportional to 12.726 $T^{1/2}$, –0.540RH, and 0.276 WSP. Decreases of ambient temperature and wind speed (when WSP < 130) will decrease $R_S$, but increases of relative humidity (up to 80%) will result in the decrease of the $R_S$.

After collection, the exposed passive samplers will be analyzed in the laboratory. The instruments and chemicals used for the analyses are basically simple and inexpensive. Ion chromatographs are for $SO_2$ and ozone passive samplers; the spectrophotometric and continuous flow analysis methods are used for $NO_2$ passive samplers; fluorometers are used for $H_2S$ passive samplers.

## Field Validation

The PASS for $SO_2$, $NO_2$, $O_3$, and $H_2S$ has been validated in many air monitoring stations in Alberta Canada for a long period of time (all seasons)[6,8,9,10,11]. The rain shelters were fastened using an outside bracket in the stations so that the passive samplers were at the same elevation as the inlet for the continuous analyzers. During validation, the temperature ranged from –40° to 35°C, the average wind speed from 20 to over 130 cm/s, and the relative humidity from 30 to 100%. The exposure times ranged from 2 days to 3 months. The correlation between the PASS and an $SO_2$, $NO_2$, $O_3$, or $H_2S$ continuous analyzer is very good.

Fig. 4 is an example of field validation. It shows a whole year comparison results of PASS $SO_2$ and an $SO_2$ continuous analyzer in Edmonton Industrial Monitoring Unit (EIMU) in Canada from June 1996 to June 1997. It can be observed that the correlation between the PASS $SO_2$ and the $SO_2$ continuous analyzers is excellent in all seasons.

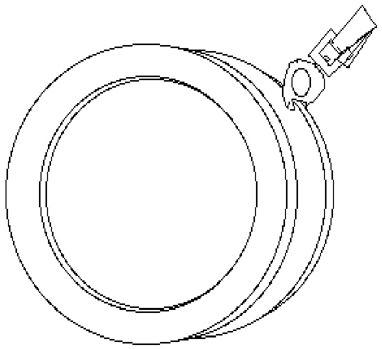

**FIGURE 4.** All season comparison results between PASS $SO_2$ and a continuously $SO_2$ analyzer in EIMU Canada from June 1996 to June 1997.

## Independent Validation

The PASS has been independently validated by many Canadian government organizations. ARC supported by NRC conducted an independent validation of the PASS $SO_2$ in 1997[12]. Two different locations at different seasons were chosen to conduct the independent validation in Alberta, Canada. Table 1 lists the locations, validation periods, $SO_2$ concentrations measured by the passive samplers and monitored by the continuous analyzers, and the relative errors in each study conducted. AEP conducted 13 months' field validation of the PASS $SO_2$, $NO_2$, and $O_3$ at several stations in Alberta, Canada from December 1998 to December 1999[13]. Those stations are located across Alberta from north to south. The AEP studies included monthly and 3-month exposures in order to compare successive monthly exposure results with 3-month exposure results at the same stations. Excellent results were obtained. Fig. 5 shows an example of the PASS $O_3$ validation results in Beaver Lodge, Canada from December 1998 to December 1999; and Fig. 6 shows the PASS $NO_2$ validation results in EIMU and Calgary residential area (CRMU). Table 2 lists comparison results between successive samplers and samplers covered the whole exposure period for PASS $SO_2$ in three locations in Alberta, Canada. It can be seen that all the comparison results are within 15% relative deviation, which indicates that PASS is a reliable system and can be used to accurately measure $SO_2$, $NO_2$, $O_3$, and $H_2S$ in air.

**TABLE 1**
**Independent Validation Results of PASS $SO_2$ by ARC**

| Location* | Exposure date | Passive ppb | Analyzer ppb | Error% |
|---|---|---|---|---|
| TMK | July 31–Aug. 28 | 1.3 | 1.4 | 7 |
| TMK | Nov. 1–Dec. 3 | 1.6 | 1.6 | 0 |
| EIMU | July 31–Aug. 28 | 1.8 | 2.0 | 10 |
| EIMU | Nov. 1–Dec. 3 | 3.0 | 3.2 | 6 |

*TMK and EIMU are locations in Alberta, Canada.

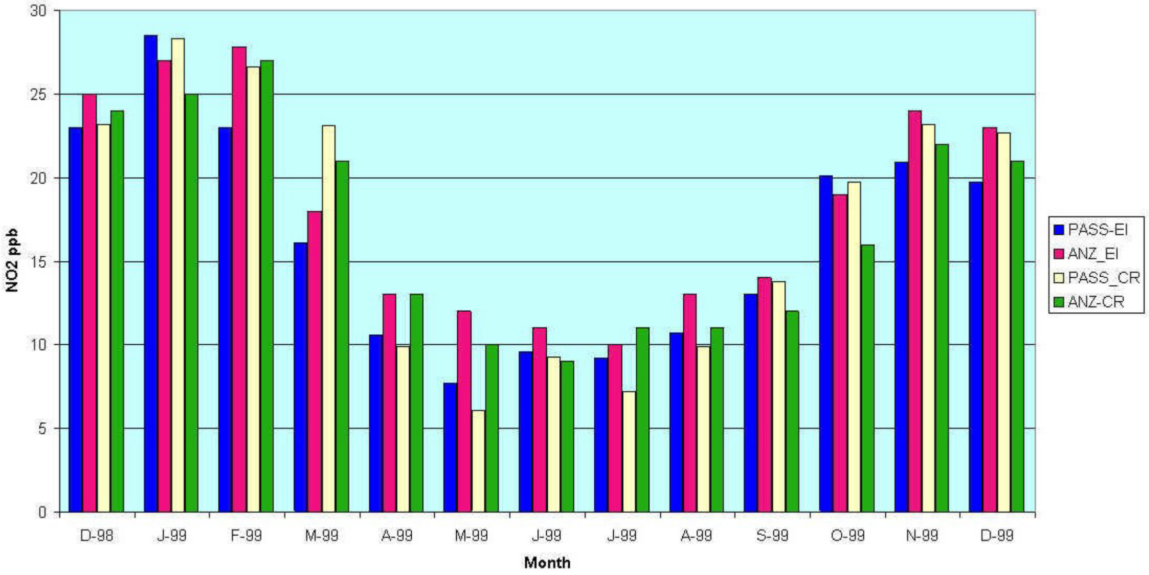

**FIGURE 5.** Validation results of PASS $O_3$ by AEP in Beaver Lodge Canada from December 1998 to December 1999 (courtesy from AEP).

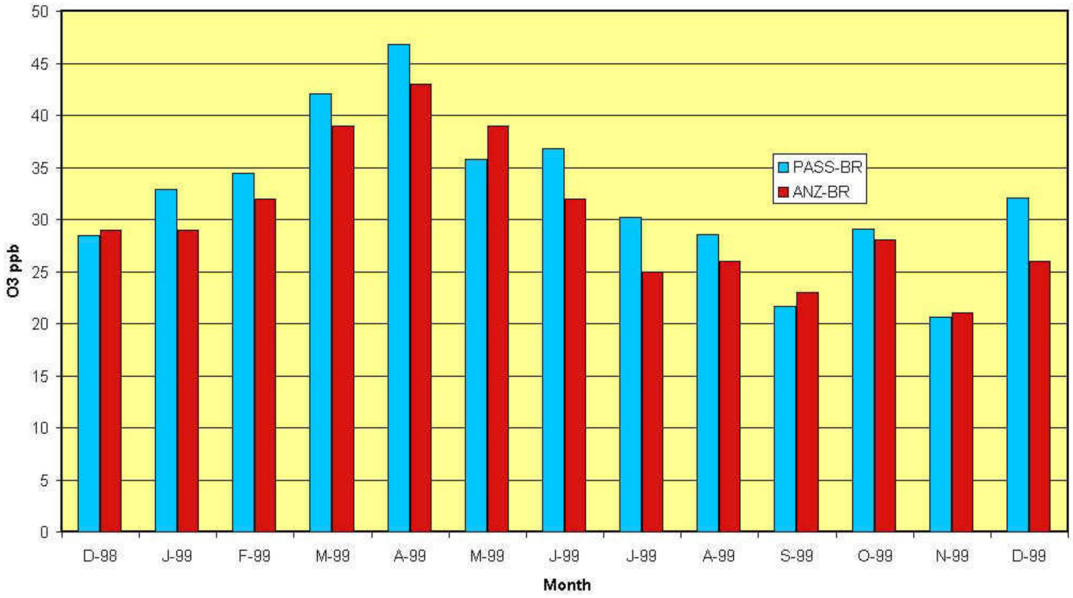

**FIGURE 6.** Validation results of PASS $NO_2$ by AEP in Edmonton and Calgary Canada from December 1998 to December 1999 (courtesy from AEP).

**TABLE 2**
**PASS $SO_2$ Results Comparison of Three Successive Monthly Exposures and Exposures Covered the Whole Three Months in Different Alberta Locations**

| Location* | Monthly $SO_2$, ppb | | | Average $SO_2$, ppb | 3-month $SO_2$, ppb | Error % |
|---|---|---|---|---|---|---|
| | June 1999 | July 1999 | August 1999 | | | |
| EIMU | 2.2 | 2.4 | 2.2 | 2.3 | 2.3 | 0 |
| BR | 0.9 | 1.2 | 1.0 | 1.0 | 1.1 | 10 |
| CIMU | 2.7 | 2.0 | 2.2 | 2.3 | 2.5 | 8 |

*EIMU – Edmonton industrial monitoring unite, Canada; BR – Beaver Lodge, Canada; CIMU – Calgary industrial monitoring unite, Canada

## Application

The PASS has been used in many national and international projects. Those projects include indoor air quality studies, industrial hygiene assessments, ambient air pollution monitoring programs, bio-monitoring programs, and model developments of dry deposition. The following is an application example.

Parkland air monitoring zone (PAMZ) is one of the largest airsheds in Canada. It covers hundreds of thousands square kilometers of lands. There are more than 30 passive stations and several continuous air monitoring stations in the airshed. Fig. 7 shows $SO_2$ concentration distribution of February 2000 in PAMZ. The right side of the map is Canada national park area. There is almost no manmade $SO_2$ pollution there. Therefore, the $SO_2$ concentrations in this area were much lower than in other areas where there were a lot of industrial activities.

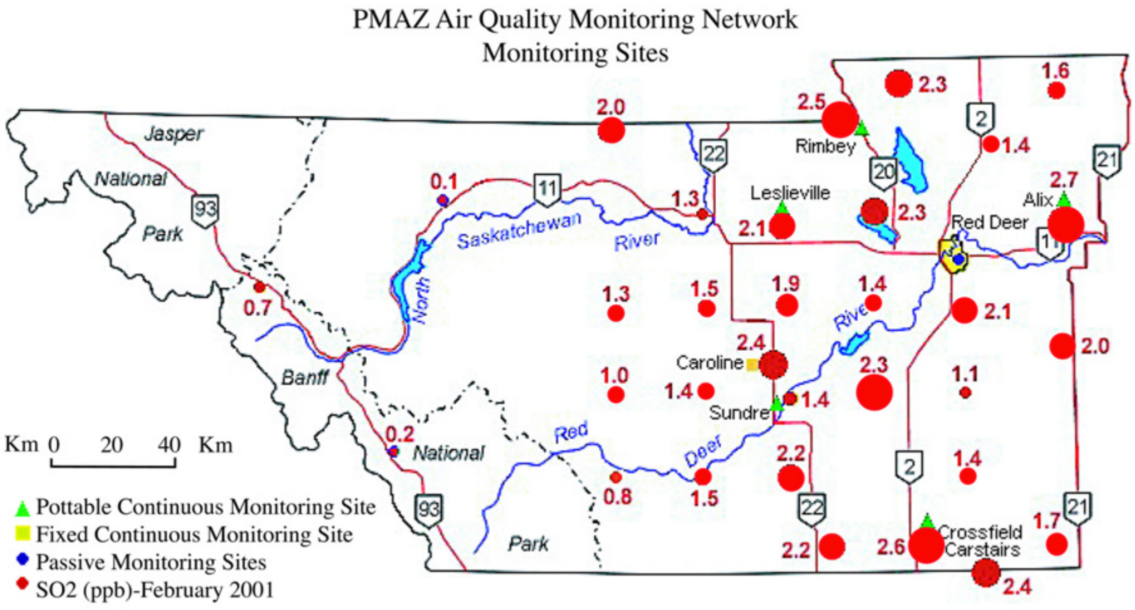

**FIGURE 7.** February 2000 $SO_2$ distribution in Parkland Air Monitoring Zone Canada (courtesy from PAMZ)

## PROPER USE OF PASSIVE SAMPLERS IN THE FIELD STUDY

Through many years of experience, we have found that the proper use of the PASS has played a very important role for correct assessment of air pollution concentrations in air. The principles of proper use of passive samplers in the field study mainly include four parts:

- How to design a study program;
- How to properly install passive samplers;
- How to determine passive air sampling rates;
- How to establish a relationship between air pollution concentrations obtained by passive samplers and air quality standards. For other special studies, such as health and vegetation studies, the problem is how to establish a relationship between air pollution concentrations and health or vegetation effects.

### Program Design and Site Selection

Passive sampling technology can be generally used for two purposes: indoor and outdoor air pollution studies. Indoor studies are basically for indoor air quality, personal exposure, and industrial hygiene purposes. Since indoor environments are relatively simpler than outdoor environments, program designs for indoor studies are easier than those for outdoor studies.

There are generally three applications for using passive sampling technology outdoors. The most common application is use as a compliance tool. In this case, the passive samplers are installed upwind and downwind of a facility to demonstrate the impact that facility has on the local environment (health issues or vegetation issues). The second application is use as additional references or supplementary parameters at a continuous or intermittent monitoring site. The final application is use in large network studies where the aim is to determine average level of pollutants impacting the area or pollutant distributions in the area. The last application has become more and more popular. There have been three air monitoring zones in Alberta, Canada. More zones are under development now. The air monitoring zone normally covers hundreds of thousands square kilometers. Many organizations from government, industry, agriculture, private sector, etc. are involved in the network operation and management.

Site selections are based on a study's need. There are differences for individual studies and network air quality studies. For individual studies, the study site should be close to the study subject. But for network air quality studies, uniform-siting criteria are necessary to ensure the collection of compatible and comparable data. The following criteria should be considered for network studies.

1. Monitoring sites should not be located near pollution sources that can cause inappropriately high measurement. For example, in farm areas, typical sources will include farm machine emission, fuel and farm equipment storage, etc. A distance of at least 100 m between the site and the source is recommended.
2. Monitoring sites should not be close to roadways. The distance between the site and the roadway should be more than 10 m.
3. In industrial areas, the site should be selected to represent a typical, not a worst case, air pollution concentration level.
4. The preferred site is flat, nonrepresentative valley locations and siting at the top or base of a hill unless special studies are needed for these locations and weather information can be obtained.
5. Monitoring site should be located away from nearby obstructions such as buildings, trees, etc. The ideal monitoring site should be more than 20 m from the nearest tree canopy defined by the drip line.

For network studies, combining passive sampling technology and real-time analytical technology is necessary. Air monitoring stations equipped with air pollution continuous analyzers and climate parameter measurement devices can provide not only weather information for the need of regional passive sampler stations but also comparison results between analyzers and passive samplers. The comparison results are necessary for the network's QA/QC program.

## Installation of Passive Samplers

For credible results, it is recommended that triplicate or at least duplicate passive samplers be used for each monitoring location. To validate results, travel blanks must be included. The installation height of the rain shelter should follow the standard site criteria or legal requirement in the study area. For example, the requirements of Alberta Environmental Protection Services[14] are as follows (Fig. 8):

- the rain shelter should be above ground 1–3 m;
- election angle should be $<30^{o}$ from the diffusion barrier surface of the passive sampler to the top of any obstacle; or
- the distance from the obstacle to the rain shelter should be >10 times the obstacle height.

In general, the rain shelter must be installed properly to prevent passive samplers from interference from animals, humans, or surroundings. If there are several rain shelters in one location, they can be installed together (Fig. 9). Passive samplers’ exposure start and end times and dates should be recorded on a field-sampling sheet.

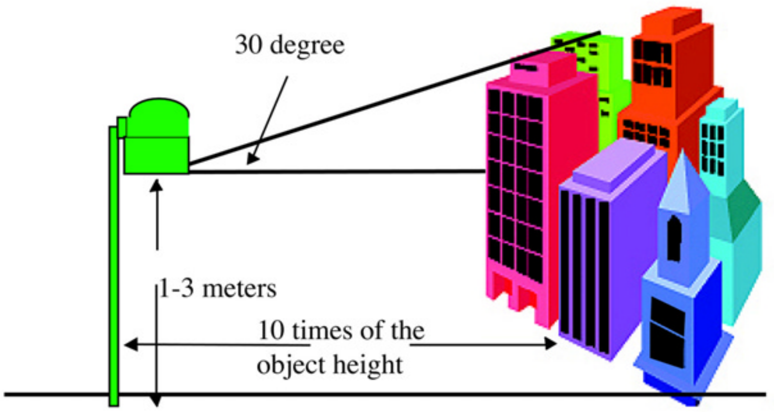

**FIGURE 8.** Illustration of passive installation criteria.

### *Passive Sampling Rate Calculation*

As discussed before, the passive sampler’s sampling rate cannot be a fixed value. It depends on many factors such as temperature, relative humidity (RH), wind direction, wind speed, sampler’s structure, and collection media. In PASS, an equation, which accounts for variations in temperature, relative humidity, and wind speed, is used to calculate sampling rates[6,8,9,10,11].

The meteorological parameters used in the sampling rate equations can be obtained from local weather forecast stations or climate normals, such as Canadian Climate Normals (CCN)[12]. CCN is a publication of the Canadian climate program average over a 30-years period of weather parameters for different locations. It is found that the parameters listed in CCN were close to the parameters

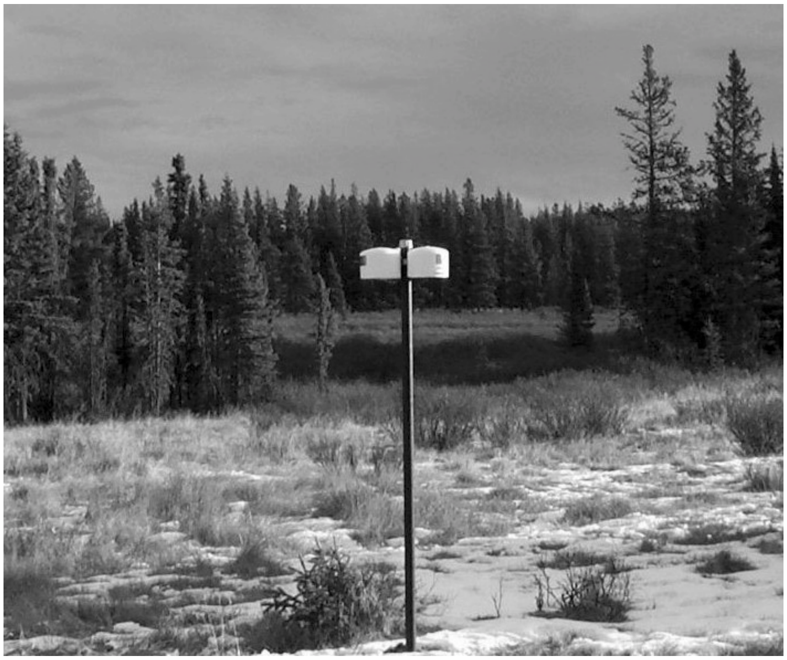

**FIGURE 9.** PASS multi-rain shelter installation (courtesy from PAMZ).

**TABLE 3**
**Comparison of Monthly Temperature and Relative Humidity in Fort. St. John Canada between Year 2000 and CCN 30 Years' Average**

| Month | Temperature C | | Relative Humidity % | |
|---|---|---|---|---|
| | **2000** | **CCN** | **2000** | **CCN** |
| January | -15 | -15 | 70 | 73 |
| February | 10 | -11 | 77 | 70 |
| March | -7 | -6 | 77 | 77 |
| April | 5 | 4 | 60 | 58 |
| May | 8 | 10 | 58 | 55 |
| June | 13 | 14 | 65 | 61 |
| July | 14 | 17 | 67 | 65 |
| August | 16 | 15 | 67 | 70 |
| September | 11 | 10 | 70 | 70 |
| October | 3 | 4 | 64 | 67 |
| November | -3 | -7 | 80 | 76 |
| December | -14 | -13 | 78 | 75 |

monitored by the local weather station. Table 3 lists meteorological parameter comparison in Fort St. John of British Columbia, Canada between 2000 and the CCN. It can be seen that the values are very close.

Actually, even a 5°C decrease of temperature can only reduce the PASS $SO_2$ sampling rate around 4%. But during different seasons (such as summer and winter in Alberta) or at different locations in the world (such as Florida, U.S. and Alberta, Canada in winter), the temperature difference might be as high as 50°C. This change is very significant. Assuming RH = 60%, WSP = 12 kmph, the high temperature is 30°C, and the low temperature is –10°C, the $SO_2$ sampling rates will change from 91 to 76 ml/min. The difference is 15 ml/min. The relative deviation is about 20% to each individual sampling rate. That change is significant.

The change of RH at 5% will also not seriously affect the sampling rate. If RH changes from 60 to 65%, the $SO_2$ $R_S$ change is about 3%. If the RH change is large, such as from 57 to 78%, combined with the temperature change from 10° to –9°C, the $SO_2$ sampling rate changes from 85 to 66 ml/min. This change is very significant (>20%).

Our studies show that when meteorological conditions are averaged over a long period such as one month in the same season, they do not vary greatly within a special geographical area (Table 3). For short period studies such as 8 or 24 h, the meteorological parameters might vary from location to location. Real measurements of meteorological parameters are needed.

### Relationship between Air Pollution Concentrations Obtained by Passive Samplers and Air Quality Standards

## CONCLUSIONS

The new Maxxam all-season passive sampling system described in this paper is suitable for monitoring $SO_2$, $NO_2$, $O_3$, and $H_2S$ concentrations in air, especially for networks. The unique design of PASS can avoid the effects of suspended particles in air and wind direction on sampling rates. The equations associated with meteorological parameters determined from the lab and field studies can provide reasonable sampling rates for field measurement. Passive sampling technology can provide accurate measurement for air pollution only when it is used properly. The proper uses of passive samplers include study program design, site selection, passive sampler installation, correct sampling rate determination, and interpretation of study results.
